# People Living With HIV Have More Intact HIV DNA in Circulating CD4+ T Cells if They Have History of Pulmonary Tuberculosis

**DOI:** 10.20411/pai.v9i2.722

**Published:** 2024-09-23

**Authors:** Marc Antoine Jean Juste, Yvetot Joseph, Dominique Lespinasse, Alexandra Apollon, Parmida Jamshidi, Myung Hee Lee, Maureen Ward, Esther Brill, Yanique Duffus, Uche Chukwukere, Ali Danesh, Winiffer Conce Alberto, Daniel W. Fitzgerald, Jean W. Pape, R. Brad Jones, Kathryn Dupnik

**Affiliations:** 1 GHESKIO Centers, Port au Prince, Haiti; 2 Department of Medicine, Weill Cornell Medicine, New York, NY

**Keywords:** HIV, tuberculosis, virus latency, proviruses, Haiti, interleukin-1, interleukin-2

## Abstract

**Background:**

A primary barrier to curing HIV is the HIV reservoir. The leading infectious cause of death worldwide for people living with HIV is tuberculosis (TB), but we do not know how TB impacts the HIV reservoir.

**Methods:**

Participants in identification and validation cohorts were selected from previously enrolled studies at Groupe Haïtien d'Étude du Sarcome de Kaposi et des Infections Opportunistes (GHESKIO) in Port au Prince, Haiti. Intact and non-intact proviral DNA were quantified using droplet digital PCR of peripheral blood mononuclear cell (PBMC)-derived CD4+ T cells. Kruskal-Wallis tests were used to compare medians with tobit regression for censoring.

**Results:**

In the identification cohort, we found that people living with HIV with a history of active pulmonary TB (n=19) had higher levels of intact provirus than people living with HIV without a history of active TB (n=47) (median 762; IQR, 183-1173 vs 117; IQR, 24-279 intact provirus per million CD4, respectively; *P*=0.0001). This difference also was seen in the validation cohort (n=31), (median 102; IQR, 0-737 vs 0; IQR, 0-24.5 intact provirus per million CD4, *P*=0.03) for TB vs no-TB history groups, respectively. The frequencies of CD4+ T cells with any detectable proviral fragment was directly proportional to the levels of interleukin-1 beta (r=0.524, *P*= 0.0025) and interleukin-2 (r=0.622, *P*=0.0002).

**Conclusions:**

People living with HIV with a history of active pulmonary TB have more HIV pro-virus in their circulating CD4+ T cells, even years after TB cure. We need to characterize which CD4+ T cells are harboring intact provirus to consider the impact of T cell-targeting HIV cure interventions for people living in TB-endemic areas.

## BACKGROUND

The primary barrier to curing HIV infection is the pool of intact HIV proviruses integrated into host cell DNA throughout the bodies of people living with HIV, called the HIV reservoir. Larger HIV reservoirs correlate with adverse HIV-related disease outcomes [1–4]. HIV reservoir size is related to peak viral load, duration of time with HIV infection, and time living with HIV prior to starting antiretroviral therapy (ART) [5]. Reservoir size has also been shown to be inversely correlated to the nadir CD4 count [6], likely a proxy for longer duration of infection. Recent studies show that timing of initiation of combination ART (cART) (early vs late) can impact not only the HIV reservoir quantity [7] but also the qualities of integrated proviral DNA — including the fraction that is intact versus fragmented [8].

Studies have compared changes in the reservoir size in response to antigen stimulation such as vaccination [9, 10], and we have data on the impact of a limited number of coinfections on the HIV reservoir. For example, cytomegalovirus and Epstein-Barr virus viremia have been associated with a decrease in the rate of decay of the HIV reservoir in men [11, 12]. The leading infectious cause of death worldwide for people living with HIV is tuberculosis (TB) [13]. One group used quantitative PCR of total HIV DNA in peripheral blood mononuclear cells (PBMC) to approximate reservoir size [3] in people in Uganda with and without active pulmonary TB and did not see a statistically significant difference [14]. However, in a cohort in China, people living with HIV with a history of TB had a higher level of circulating HIV DNA in PBMC than people without TB, but they also had a higher viral load pre-ART [15]. The association of increased HIV viral load and active TB has been described [16, 17] and is a potential confounder of the latter reported association of TB with reservoir size.

There are multiple assays to measure the HIV reservoir in CD4+ T cells [18], but the emerging gold standard is the intact proviral DNA assay (IPDA), which distinguishes intact from defective proviruses [19, 20]. We used a modified IPDA [21] to measure intact and fragment HIV provirus in circulating CD4+ T cells of people living with HIV in a resource-limited setting with a TB syndemic. Our objective was to determine how quantities of CD4+ T cells containing HIV provirus vary in people with a history of active bacteriologically confirmed pulmonary TB disease concurrent with or after diagnosis of HIV. Because of the overall worse outcomes for people living with HIV who have a history of TB [22], we hypothesized that proviral load would be higher in people living with HIV with a history of TB.

## METHODS

### Study Site

The prevalence of HIV in adults in Haiti was 1.7% in 2023 [23]. Haiti has the highest rate of TB in the Americas. In 2022, there were 154 new cases of TB per 100,000 people, and 15% of those individuals were living with HIV [24]. The Haitian Group for the Study of Kaposi's Sarcoma and Opportunistic Infections (with the French acronym of GHESKIO for Groupe Haïtien d'Étude du Sarcome de Kaposi et des Infections Opportunistes) in Port au Prince, Haiti, is a Haitian non-governmental organization that was founded in 1982 and is the largest provider of HIV and TB care in the Americas [25].

### Research Ethical Approval and Clinical Standard of Care

This study was approved by institutional review boards at Weill Cornell Medicine (protocols 1401014658 and 1612017803) and GHESKIO. All study participants provided written informed consent [26]. There was no difference in clinical care between participants who did or did not decide to participate in either of these studies. All participants were offered ART and anti-TB treatment and continuing care for HIV and TB per the Haitian national guidelines and the GHESKIO standard of care at the time, which adhered to World Health Organization recommendations. All people at GHESKIO who are diagnosed with HIV and do not have active TB are prescribed preventive therapy for treatment of presumptive latent TB because of the high rate of TB in the community, and prior studies at GHESKIO that have documented the survival benefit of universal TB preventive treatment for people living with HIV. [27]

### Identification Cohort

For the identification cohort, we designed a nested case-control study from a parent cohort study of TB called the Tri-institutional Tuberculosis Research Unit (TBRU) [28–30] (Supplementary Figure 1, Supplemental Methods). TB was defined as pulmonary TB that had been bacteriologically confirmed by positive sputum TB GeneXpert, positive sputum smear for acid-fast bacilli, or sputum culture that grew *Mycobacterium tuberculosis*. Latent TB testing was performed with the Quantiferon-TB Gold In-Tube interferon-gamma release assay (IGRA) (Qiagen). Blood was collected using heparin as an anticoagulant for the isolation of plasma and PBMCs.

Participants for the case-control study were recruited between June 2015 and January 2019 during which time their PBMCs were collected and cryopreserved.

### Validation Cohort

For the validation study, we used PBMC collected as part of a parent case-control study of TB recurrence in people living with HIV (Supplementary Figure 2, Supplementary Methods). Each episode of TB was bacteriologically confirmed by positive sputum TB GeneXpert, positive sputum smear for acid-fast bacilli, or sputum culture that grew *M. tuberculosis*. Each participant had completed treatment for TB at least 6 months prior to study enrollment. Recurrent TB was defined as at least 2 episodes of bacteriologically confirmed TB, with the second episode occurring at least 6 months after completing treatment for the first episode. Latent TB testing was performed using the Quantiferon-TB Gold Plus In-Tube interferon-gamma release assay (IGRA) (Qiagen). Blood was collected using EDTA as an anticoagulant for the isolation of plasma and PBMCs. The nested case-control study included all study participants with adequate PBMC available in New York as of October 2021. We calculated that 13 people in the TB group and 13 people in the no-TB group would give 90% power to detect the difference in proviral loads between the TB and no-TB groups seen in the identification subcohort, with a standard deviation of 500. Study participants were enrolled and PBMCs were collected between December 2019 and October 2020.

### Preparation of Peripheral Blood Mononuclear Cells

For both studies, PBMCs were isolated at GHESKIO using density gradient separation (Ficoll-Hypaque, GE Healthcare) with 5x10^6^ PBMC aliquots frozen and then shipped to New York where they were stored at -135C. PBMC specimens were identified with a unique patient identifier that did not correspond to their TB status. Participants were assigned to TB and no-TB groups after laboratory assessments were complete.

### Intact Proviral DNA Assay

For each participant, a single aliquot of 5x10^6^ PBMC was thawed and washed before CD4+ T cell isolation by negative selection (EasySep™ Human CD4+ T cell Enrichment Kit, Stem Cell Technologies). DNA was then isolated using PureGene (Qiagen) for the identification cohort and DNeasy Miniprep (Qiagen) for the validation cohort. Intact proviral DNA amplification was determined using droplet digital PCR (ddPCR) on a QX200 instrument (Bio-Rad) [19]. Amplification of the HIV *psi* and *env* regions was performed independently in parallel with quadruple technical replicates. The degree of DNA shearing was assessed using *rpp30.* Samples with PCR reactions with fewer than 10,000 droplets read were excluded from the calculation for that participant. Samples that did not have any detectable *env* or *psi* amplification using the primers and conditions described in Bruner et al [19] and Gunst et al [31] were re-run with secondary *env* primers and probes as described in Kinloch et al [21]. The rates of primer usage by group are included in Tables 1, 2, and 3. Samples with no amplifiable HIV DNA (*env* fragment and *psi* fragment) with both primer sets were also excluded. From the ddPCR, we generated numbers of 3′ defective (*env*), 5′ defective (*psi*), and presumed intact provirus containing both *env* and *psi* per million CD4+ T cells.

**Table 1.** Demographics of the Identification Cohort

	TB (n=50)		No TB (n=50)		
	Untreated TB (n=25)	History of TB or on treatment (n=25)	IGRA-positive (n=25)	IGRA-negative (n=25)	*P-* value^*^
Sex (#female [%])	10 (40%)	14 (56%)	16 (64%)	17 (68%)	0.07
Age in years (mean +/− SD)	38.16 +/− 7.5	38.8 +/− 6.8	44 +/− 5.9	44 +/− 4.3	0.0001
Time since HIV dx in years (median [IQR])	0.02 (0.02-0.03)	3.1 (1.1-5.4)	12.8 (11.7-14.3)	11 (10.5-14.7)	0.0001
Time on ART in years (median [IQR])	0	2.9 (0.9-4.5)	10 (8.7-10.7)	8.9 (8-10.2)	0.0001
Time between diagnosis and ART (median [IQR])	.06 (0.02-0.07)	0.9 (0.4-.57)	2.7 (2.3-4.1)	2.6 (1.3-4.7)	0.0001
Alternate IPDA primers used (number [%])	7 (28%)	9 (36%)	4 (16%)	3 (12%)	0.033
Time since last diagnosis of TB in years (median [IQR])	0.01 (0.005-0.016)	2.37 (0.88-4.38)			
Time between diagnosis of HIV and last diagnosis of TB in years (median [IQR])	0.011 (0.005-0.014)	0.049 (0.01-1.17)			

* *P*-value is the comparison between TB group (n=50) and no-TB group (n=50) using chi-square (sex), two-sided *t* test (age), or Mann-Whitney test for non-normally distributed data.

**Table 2.** Demographics of the Final Analyzed Group in the Identification Cohort

	TB (n=19)	No TB (n=47)	*P*-value
Sex (#female [%])	11 (58%)	30 (63%)	0.66
Age (mean [SD])	40 (33-47)	44 (39-49)	0.005
Time since HIV dx in years (median [IQR])	4.3 (2.3-5.8)	12.5 (10.6-14.6)	0.0001
Time on ART in years (median [IQR])	3 (2-5)	9.3 (8.2-10.4)	0.0001
Time between diagnosis and ART (median [IQR])	0.13 (0.04-0.9)	2.6 (1.6-4.2)	0.0001
Alternate IPDA primers used (number [%])	9 (47%)	7 (15%)	0.006
Time since last diagnosis of TB in years (median [IQR])	3.2 (1.18-4.53)		
Time between diagnosis of HIV and last diagnosis of TB in years (median [IQR])	0.112 (0.01-1.76)		

**Table 3.** Demographics of the Final Analyzed Group in the Validation Cohort

	TB (n=18)	No TB (n=13)	*P*-value
Sex (#female [%])	10 (55%)	7 (54%)	0.9250
Age (mean [SD])	44 +/− 10.1	43.3 +/− 12.1	0.8626
Time since HIV dx in years (median [IQR])	6.7 (5.4, 7.8)	6.4 (4.7, 7.9)	0.8101
Time on ART in years (median [IQR])	6.2 (4.1, 7)	5 (4, 6.5)	0.8727
Time between diagnosis and ART (median [IQR])	0.1 (0, 0.2)	0.8 (0, 1.6)	0.0722
HIV viral load (median, [IQR])	0 (0-518)	0 (0-77)	0.2941
IGRA positivity (# positive [%])	9 (50%)	10 (77%)	0.129
Alternate IPDA primers used (number [%])	4 (22.2%)	3 (23%)	0.9560
Time since last diagnosis of TB in years (median [IQR])	3.2 (2.3, 5.0)		
Time between diagnosis of HIV and last diagnosis of TB in years (median [IQR])	2.3 (0.03, 4.96)		
Number with recurrent TB (%)	7 (38%)		

### HIV Viral Load

None of the participants in the identification cohort had detectable HIV in their plasma, but since blood samples from participants in the identification TBRU study were collected with heparin as the anticoagulant, viral load results were potentially not reliable [32]. Therefore, we also tested plasma for circulating HIV p24 GAG protein using an enzyme-linked immunosorbent assay (RETRO-TEK™ HIV-1 p24 Antigen ELISA, ZeptoMetrix) with immune dissociation and reactive confirmation (HIV-1 p24 ICx/CRx kit, ZeptoMetrix). Participants who had detectable p24 or who did not have plasma available for p24 testing were excluded from final analysis of the identification cohort.

Participants in the TB Recurrence study from which the validation cohort was selected were to have had undetectable viral loads prior to enrollment in the study. However, because of the impact that active HIV replication could have on the IPDA, we measured HIV load as well as p24 in these participants. Plasma was centrifuged at 10,000*g* for 10 minutes to remove particulates. RNA was extracted from plasma using a semi-automated method, and HIV viral load was quantitated using a previously described integrase single copy assay [33, 34]. Samples were analyzed on an ABI Viia7 Real-Time PCR System (Thermo Fisher Scientific). Cycle threshold values were compared with a validated HIV RNA standard run to determine concentration. Participants who had a viral load greater than 1,000 copies/mL and detectable p24 after immune dissociation and neutralization were excluded.

### Cytokine Levels

Plasma levels of granulocyte-macrophage colony-stimulating factor (GM-CSF), interferon gamma (IFN-γ), interleukin-1 beta (IL-1β), interleukin-2 (IL-2), interleukin-4 (IL-4), interleukin-5 (IL-5), interleukin-6 (IL-6), interleukin-12p70 (IL-12p70), interleukin-13 (IL-13), interleukin-18 (IL-18), and tumor necrosis factor (TNF) were measured using a bead-based multiplex assay (Th1/Th2 11-plex Human ProcartaPlex Panel, Invitrogen). Quantitation was performed using a Luminex MAGPIX system with xPONENT v4.3 software (Luminex) according to the manufacturer's instructions with the following specifications. Plasma was centrifuged at 10,000*g* for 10 minutes to remove particulates prior to aliquoting onto the Luminex plate. Samples were incubated overnight at room temperature. All samples were run in duplicate, and the average reading was used for quantitation with the standard curve. For samples that were less than the lower limit of detection, we imputed a value of half the difference between zero and the lowest concentration standard on the standard curve for each cytokine. Cytokines that were less than the level of detection for more than 50% of plasma samples did not continue to statistical analyses.

### Data and Statistical Analysis

Demographic and clinical characteristics were expressed as numbers and percentages with inter-quartile range given for continuous variables. Time since diagnosis of HIV, time between diagnosis and ART, and time since diagnosis of last episode of TB were converted from days to years for analysis. All statistical analysis was done in Stata (version 18). Distributions of variables were tested using Skewness/Kurtosis tests for normality. For normality test *P*< 0.05, Kruskal-Wallis tests were used to compare medians. Medians are reported with interquartile range (25th percentile – 75th percentile) as the indicator of variation in non-normally distributed data. Two-sided *t* tests were used for comparison of means when the normality test *P*>0.05. For log_10_ analyses, we added 1 to the proviral load so that the proviral loads of zero would not be undefined (log_10_0 is undefined whereas log_10_1 is zero). When possible, we used the unmodified provirus quantification for statistical calculations. When there was a large number of undetectable proviral loads, we utilized tobit regression for left censoring of proviral loads that were less than the lower limit of detection with the IPDA [35]. For the validation cohort analysis, we completed 2 multivariate regression analyses for factors known to be associated with proviral load and which approached a statistically significant difference between the cases and controls. We used Pearson's correlation coefficient (<pwcorr> in Stata) to compare quantities of provirus and cytokines. For the cytokine analyses, we applied a Bonferroni correction for multiple comparisons using the number of analyzable cytokines as the correction factor. For all other analyses, the cutoff for significance was *P*≤0.05.

## RESULTS

Using IPDA, we measured intact provirus, *psi* fragment, and *env* fragment levels in CD4+ T cells of 100 people living with HIV who had participated in a TB study at GHESKIO Centers in Port au Prince, Haiti. The nested cohort consisted of 25 people with active TB, 25 people with a history of active TB, 25 people with no history of TB and positive IGRA, and 25 people with no history of TB and a negative IGRA. (Table 1, Supplementary Figure 1). This resulted in 50 people in the TB group (25 active + 25 past) and 50 people in the no-TB group (25 IGRA positive + 25 IGRA negative). Despite incidence density matching, the groups had statistically sigificant differences in several factors because of the demographics of the original TBRU cohort (older no-TB controls with a longer time with HIV).

**Figure 1.**
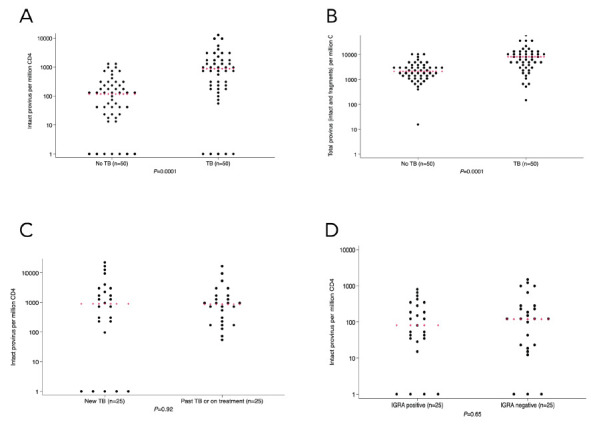
In the identification cohort (n=100) there was a larger number of circulating CD4+ T cells with intact (1A) and total (1B) provirus in people living with HIV with TB or TB history. There was no statistically significant difference in intact provirus levels when comparing people with active vs history of TB (1C). There was also no statistically significant difference in intact provirus levels between people with no history of TB with reactive vs non-reactive TB interferon-gamma release assay (1D). Kruskal-Wallis was used for statistical testing of non-normally distributed data. Red pluses indicate medians.

Within the total cohort, there was a greater percentage of CD4+ T cells containing intact provirus in people with current TB or a history of TB vs no TB (median 881 (IQR, 205-2060) vs 116 (IQR, 24-279) copies per million CD4+ T cells, respectively; *P*=0.0001) (Figure 1A). Tobit regression of detectable intact provirus also showed a statistically significant difference in intact provirus (*P* < 0.0001) between the TB and no-TB groups. The quantity of *psi* defective, *env* defective, total defective, and any viral fragment levels in CD4+ T cells was also statistically significantly higher in the TB group compared to the no-TB group (Figure 1B, Supplementary Figure 3A-C). There was no statistically significant difference in intact provirus between people with newly diagnosed untreated TB (n=25) vs history of TB or TB currently being treated (n=25) (median 897; IQR, 205-3351 vs 865; IQR, 248-1560) copies per million CD4+ T cells, respectively; *P*=0.92; Figure 1C). In the people with no history of TB, the amount of intact provirus in CD4+ T cells was not statistically significantly different by IGRA status (median 80; IQR, 35-255 for IGRA-positive vs 118; IQR, 21-279 for IGRA-negative per million CD4+ T cells,, *P*=0.65; Figure 1D). Since there was a statistically significant higher rate of alternate primer usage for IPDA in the TB vs no-TB group (32% vs 14%, respectively), we completed a post-hoc analysis where we stratified by primer type and compared intact proviral load between TB and no-TB groups. The difference in proviral load by TB history status was statistically significant in samples that were amplified with the original primers [19] (*P*=0.0012) and alternate primers [21] (*P*=0.016).

**Figure 2.**
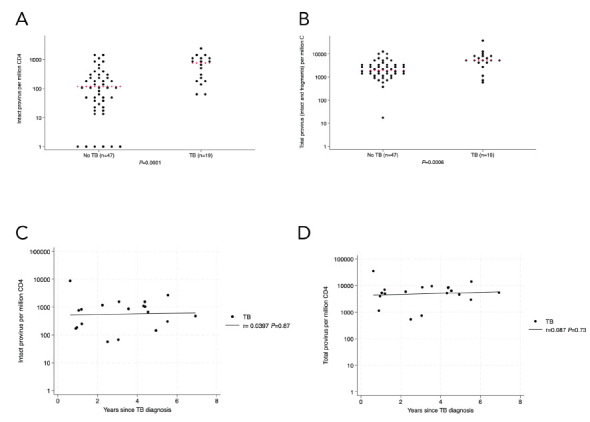
In the analyzed identification cohort subset (n=66), there was a larger number of circulating CD4+ T cells with intact HIV provirus (2A) and total provirus (2B) in people living with HIV with TB or TB history. There was no statistically significant association between intact (2C) or total (2D) provirus in circulating CD4+ T cells and length of time between the last episode of TB and enrollment in the study. Kruskal-Wallis was used for statistical testing of non-normally distributed data. Red pluses indicate medians. Correlations are Pearson's correlation coefficients.

To limit potential confounders on the difference in intact provirus observed in the initial groups, we excluded 27 people who had been diagnosed with HIV less than one year before PBMC collection. We excluded 3 people with no plasma available for p24 testing and 3 people with detectable plasma p24. One person who had TB prior to HIV diagnosis was also excluded. All other participants had TB coincident with or after diagnosis of HIV. After these adjustments (Table 2), the difference in intact proviral load between TB and no-TB groups remained statistically significant (median 762; IQR, 183-1173 vs 117; IQR, 24-279 intact provirus per million CD4+ T cells, respectively; *P*=0.0001) by Kruskal-Wallis test as well as by tobit regression (*P*<0.0001, Figure 2A). Proviral *psi* and *env* fragments and total proviral DNA were also statistically significantly higher in the TB group (Figure 2B, Supplementary Figure 3D-F). There was no association between intact (r= -0.0397, *P*=0.87, Figure 2C) or total (r=0.087, *P*=0.73, Figure 2D) provirus in CD4+ T cells and the time since TB diagnosis in the TB group.

For the validation study, we began with 34 participants from a study of TB recurrence. One participant's PBMC did not have enough live cells after thawing to have CD4+ T cells selected and DNA extracted and was therefore excluded from downstream analyses. One DNA sample failed quality control with no detectable intact *env* fragment or *psi* fragment with all tested primer sets and was therefore excluded. One person had HIV viral load >1,000 copies/mL and detectable p24 after dissociation. This person was excluded as the high-level viremia could have rendered the IPDA quantitation inaccurate.

**Figure 3.**
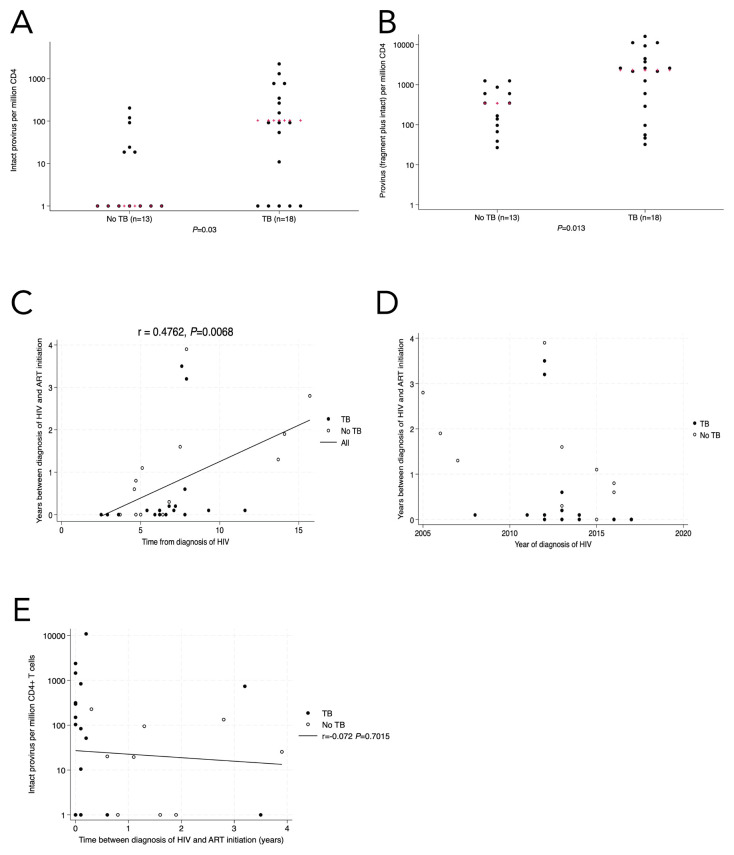
In the validation cohort (n=31), there was a higher level of intact (3A) and total provirus (3B) in the TB group compared with people with no history of active pulmonary TB. When completing multivariate analysis, we found a statistically significant correlation between time since HIV diagnosis (x-axis) and the duration of time between HIV diagnosis and ART initiation (y-axis) (3C), which was likely related to changing recommendations of when to start ART relative to CD4+ T cell count (3D). There was no statistically significant correlation between time without ART and proviral load (3E). Kruskal-Wallis was used for statistical testing of non-normally distributed data. Red pluses are the medians. Correlations are Pearson's correlation coefficients.

In the final analysis group from the validation cohort (Supplementary Figure S2, Table 3), the quantity of intact provirus was higher in the TB group compared with the no-TB group (median 102; IQR, 0-737 vs 0; IQR, 0-24.5) intact provirus per million CD4+ T cells, respectively *P*=0.03, Figure 3A). The quantity of total provirus (intact + fragment) was also higher in the TB group compared with the no-TB group (*P*=0.013, Figure 3B), although the differences in fragment pro-virus were not statistically significant between the groups (Supplementary Figure 3G-I). We also analyzed the intact provirus levels using a tobit regression model with 12 left-censored observations for intact proviral loads less than the lower limit of detection and the difference remained statistically significant (*P*=0.04).

We conducted multivariable analyses to account for the potential confounders affecting the relationship between a history of TB and intact proviral load. When sex was included in the model as an independent variable [36, 37], the association between TB and proviral load remained statistically significant (*P*=0.033 for intact provirus, *P*=0.015 for total provirus)*.* When time since HIV diagnosis and initiation of ART and time between HIV diagnosis and the initiation of ART were included in a model of the association between history of TB and intact proviral load, the association remained statistically significant (*P*=0.019 and *P*=0.035, respectively). This was also true with total (intact and defective) provirus as the dependent variable (*P*=0.018 and *P*=0.039, respectively). Because the correlation between time with HIV and interval between HIV and ART was significant, we did not generate a multivariate model that included both factors. As the time since HIV diagnosis increased, so did the time between diagnosis and initiation of ART (r = 0.476, *P*=0.007, Figure 3C), reflecting the evolving recommendations regarding the ideal timeframe to initiate ART over the past 20 years (Figure 3D) [38, 39]. There was no statistically significant correlation between quantity of intact provirus and time interval between HIV diagnosis and ART initiation (r=-0.097, *P*=0.433; r=0.016, *P*=0.896, Figure 3E).

**Figure 4.**
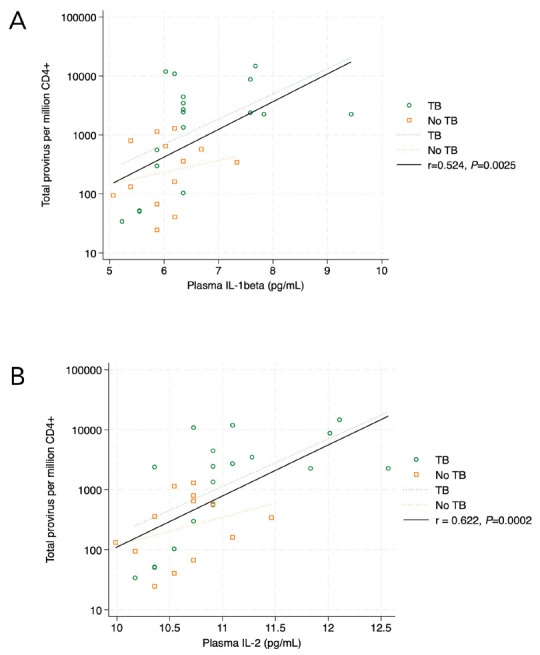
In the validation cohort, plasma IL-1β levels (4A) correlated with circulating CD4+ T cells containing HIV *psi* and/or *env* (intact + fragment) (r = 0.524, *P*=0.0025, 4A). The same association was found for plasma IL-2 levels and circulating CD4+ T cells containing HIV *psi* and/or *env* (intact + fragment) (r = 0.622, *P*=0.0002, 4B). Correlations are Pearson's correlation coefficients.

There was no statistically significant correlation between intact provirus and plasma cytokine levels for the validation cohort. However, we found that the frequency of CD4+ T cells with any detectable provirus (intact + fragment) was directly proportional to the levels of IL-1β (r = 0.524, *P*=0.0025), IL-2 (r = 0.622, *P*=0.0002), IL-12p70 (r = 0.491, *P*=0.0093), and IL-13 (r = 0.4769, *P*=0.0067). After Bonferroni correction for the 8 analyzable cytokines, (cutoff *P*_adj_=0.05/8=0.006), the association between plasma IL-1β (Figure 4A) and plasma IL-2 (Figure 4B) with CD4+ T cells containing HIV *env* or *psi* remained statistically significant.

## DISCUSSION

Using IPDA, we documented significantly higher levels of intact, fragment, and total HIV proviruses in circulating CD4+ T cells of people living with HIV and a history of bacteriologically confirmed pulmonary TB. The larger percentage of CD4+ T cells containing intact HIV provirus in people with a history of TB was shown in an identification cohort and confirmed in a validation cohort. The statistically significant difference persisted when controlling for important potential confounders of increased proviral load including time since HIV diagnosis, interval of time between HIV diagnosis and ART initiation, and sex. Interestingly the percentage of CD4+ T cells containing intact provirus was not correlated with time since last TB diagnosis; this suggests that differential reservoir size persists even after TB cure, although longitudinal studies are needed to assess this hypothesis.

T cells differentiate and expand differently in latent and active TB [40], with much higher levels of activated CD4+ T cells during active TB. Studies using PBMC from people with latent TB without HIV have found CD4+ T cells with common T cell receptor-beta sequences for different *M. tuberculosis* antigenic epitopes in people who do vs do not progress to active TB [41]. Characterization of CD4+ T cell phenotype and antigen specificity in people with HIV at various stages of HIV found more clonality of T cells and infecting provirus during later stages of HIV. In a study of T cell receptors of HIV-infected CD4+ T cells from 17 people, they found that 2 people had common anti-*M. tuberculosis* epitopes on CD4+ T cells, but that *M. tuberculosis*-specific CD4+ T cells were not over-represented in the overall p24+ CD4+ T cell pool [42]. While our results indicate a higher intact and total proviral load in people with a history of pulmonary TB disease, future studies will include characterization of which types of CD4+ T cells and which antigen specificities contain provirus in their DNA [43]. A 2010 study concluded that *M. tuberculosis-*specific CD4+ T cells were more likely to be infected by HIV, but were also more likely to be depleted during the subsequent course of infection [44]. The responsiveness to *M. tuberculosis* was based on cell surface marker phenotypes and cytokine production in reponse to *M. tuberculosis* “antigens.” Newer methodologies will allow us to determine CD4+ T cell reactivity at the level of the T cell receptor epitope in cells infected with HIV.

Local inflammation can increase HIV replication and viral load at sites where HIV and replicating *M. tuberculosis* exist, even prior to the development of symptomatic TB [45]. The increase in inflammation in people with latent TB that precedes clinically apparent active TB disease [46] may drive the increase in the HIV reservoir by activating latently infected cells or generating more activated cells that are at risk for infection by circulating HIV. The existing literature is inconclusive about a correlation between HIV provirus and inflammation [37, 47, 48] and TB and inflammation [49–51]. This study's contribution is in demonstrating a correlation of total provirus with larger plasma concentrations of pro-inflammatory cytokines IL-1β and IL-2. Production of these cytokines may drive HIV activation or be a response to HIV [52]; they are also important in the balance between control of *M. tuberculosis* infection and pathology related to immune response to *M. tuberculosis.* [53]

A strength of this study is the assessment of intact provirus in CD4+ T cells of people living with HIV in a resource-limited setting where TB is syndemic. The use of IPDA to quantitate provirus from a relatively small volume of starting material (5x10^6^ PBMC) meant that PBMC collected from standard phlebotomy during a research cohort study could be leveraged. This has important implications for how people can use IPDA to study HIV reservoir in a variety of research and clinical settings. Study limitations include incomplete nadir CD4+ T cell count and HIV viral load testing due to the changing landscape of CD4+ T cell and viral load testing in HIV care and the variability in the availability of this test at GHESKIO. This study only included people with bacteriologically confirmed pulmonary TB in order to have a TB group with highest certainty for having had TB. However, we do not know how these results will be generalizable to people treated empirically for TB or with extrapulmonary TB. We hypothesize that we will see the same increase in circulating CD4+ T cell HIV reservoir, but that study needs to be conducted. We note that the prevalent HIV subtype in Haiti is subtype B, and that this study needs to be replicated in communities where subtypes other than subtype B are prevalent. In addition to differences in techniques for quantitating HIV provirus, different HIV subtypes might explain the discrepant results regarding association between HIV DNA levels and TB seen in studies from China, Uganda, and now Haiti [14, 15].

We used 2 sets of primers to amplify HIV provirus from CD4+ T cells, which is consistent with other studies using clinical samples from non-US locations [21]. The percentage of DNA in the TB and no-TB groups needing alternate primers for amplification was statistically significantly different in the identification cohort, which may reflect different times of HIV acquisition in the TB and no-TB cohorts. When comparing proviral load obtained using original or alternate primers, the association between TB and HIV proviral load remained statistically significant. The median intact proviral loads were approximately 7-fold higher in the TB group compared with the no-TB group in both the identification and validation cohorts. However, the median intact proviral loads for the groups differed between the identification and validation cohorts. This may be because of differences in the parent studies for the identification and validation cohorts or that the testing of the identification and validation cohorts was separated by over a year which meant different reagent lot numbers, different technicians running the assay, and a different standardized DNA extraction method in use in the lab. We believe this underscores the importance of running appropriate controls for every set of IPDA experiments.

In the identification cohort, we did not see a statistically significant difference in intact provirus between IGRA-positive and IGRA-negative people living with HIV who did not have a history of active TB. Everyone diagnosed with HIV at GHESKIO is treated empirically for latent TB, so all of these participants would have received isoniazid since diagnosis of HIV [27]. IGRA reversion rates after latent TB treatment are not indicative of treatment success or failure [54]. Participants in these 2 cohorts live in a region where the rate of new diagnosis of pulmonary TB was 5% in a recent community-level screening [55] with a rate of 70% IGRA positivity in people without HIV [28]. Therefore, regardless of the result of the IGRA, we know that study participants in care at GHESKIO have a high baseline level of exposure to *M. tuberculosis*. Participants in the validation cohort TB and no-TB groups were well-matched with regard to age, sex, duration of HIV, and time between HIV diagnosis and ART initiation, with one group mounting an *ex vivo* interferon gamma response to *M. tuberculosis*-specific peptides as measured by IGRA and the other not. Therefore, we suggest that the development of and survival after active TB disease may be the driver of increased provirus load in circulating CD4+ T cells, rather than *M. tuberculosis* infection, per se. Of note, the participants in the identification cohort were tested for latent TB using the Quantiferon Gold assay, which did not yet include a peptide panel optimized for recognition by CD8+ T cells.

## CONCLUSION

The increased risk of TB for people living with HIV does not normalize to that of the general population even when HIV is virologically suppressed and CD4+ T cell counts have recovered. People with TB have higher all-cause mortality even after TB is cured, and people with higher HIV reservoir have worse clinical outcomes. Therefore, we hypothesized that HIV reservoir size would be higher in people with a history of TB, and this is supported by our data showing increased intact and total provirus in circulating CD4+ T cells of people with a history of bacteriologically confirmed active pulmonary TB in 2 different cohorts in Haiti. TB and other coinfections should be considered when studying the dynamics of the HIV reservoir, particularly when evaluating candidate HIV cure strategies.

## SUPPLEMENTARY DATA

Supplementary materials are available at the *Pathogens and Immunity* website. Supplementary data may be provided by the authors to benefit the reader. Supplementary data are not copyedited and are the sole responsibility of the authors. Questions or comments related to supplementary materials should be addressed to the corresponding author.
