# Supplementary material for: People Living With HIV Have More Intact HIV DNA in Circulating CD4+ T Cells if They Have History of Pulmonary Tuberculosis
Source: Pathog Immun. 2024 Sep 23;9(2):172–93. doi: 10.20411/pai.v9i2.722 (PMC11432494; doi:10.20411/pai.v9i2.722)
Supplement: Supplemental Figures 1-3 [file pai-9-172-s01.pdf]

PATHOGENS AND IMMUNITY
IN ASSOCIATION WITH CASE WESTERN RESERVE UNIVERSITY AND THE RICHARD J. FASENMYER FOUNDATION

Supplementary Figure 1

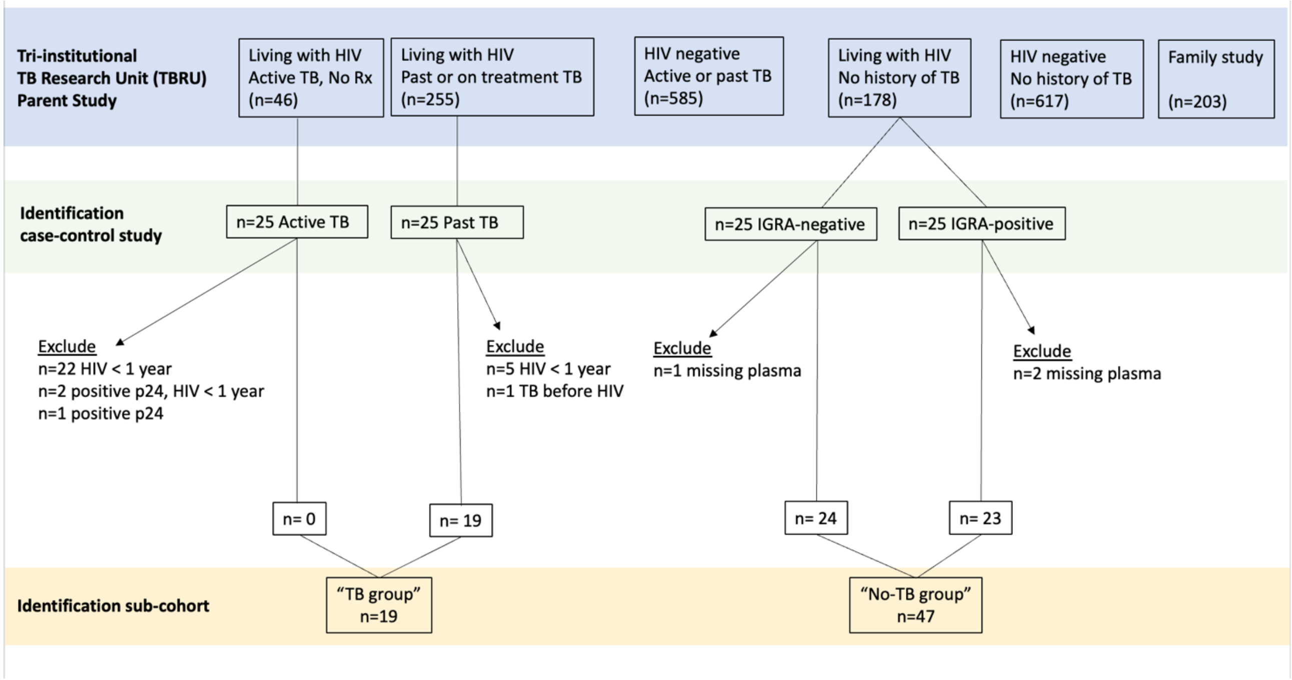

Supplemental Figure 1. Flow chart showing the parent cohort and nested case-control study for the identification cohort.

Supplementary Figure 2

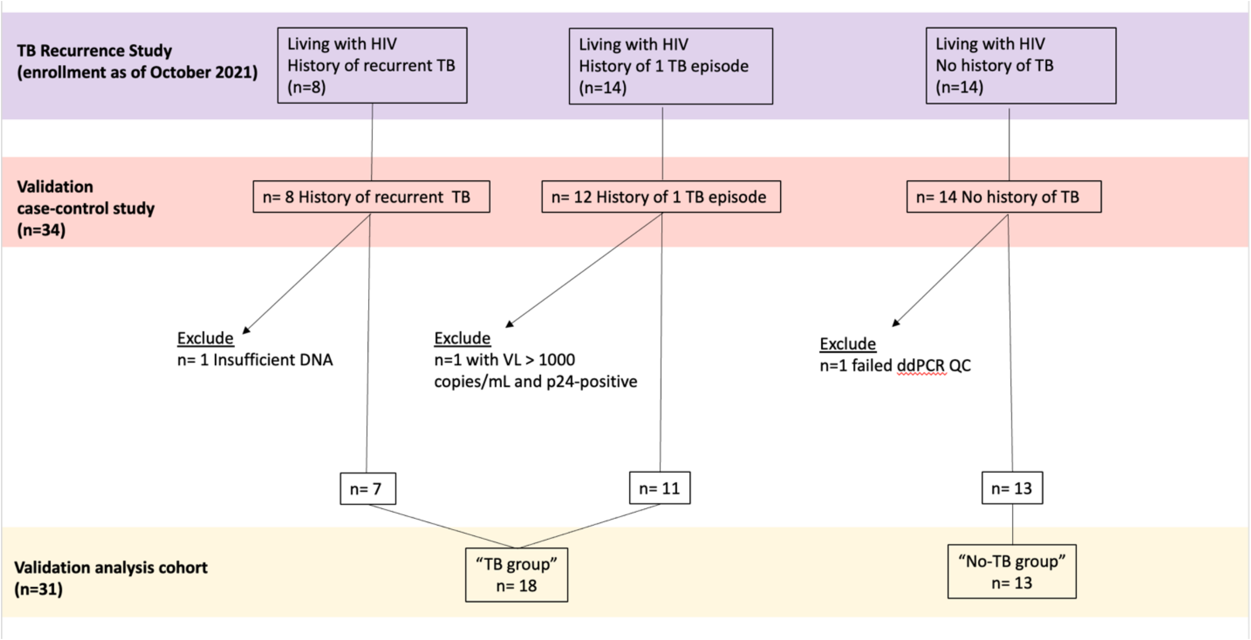

Supplemental Figure 2. Flow chart showing the parent cohort and case-control study for the validation cohort.

Juste MAJ, Joseph Y, Lespinasse D, Apollon A, Jamshidi P, Lee MH, Ward M, Brill E, Duffus Y, Chukwukere U, Danesh A, Alberto WC, Fitzgerald DW, Pape JW, Jones RB, Dupnik K. People Living With HIV Have More Intact HIV DNA in Circulating CD4+ T Cells if They Have History of Pulmonary Tuberculosis. *Pathogens and Immunity*. 2024;9(2):172–193. doi: 10.20411/pai.v9i2.722

PATHOGENS AND IMMUNITY
In Association with Case Western Reserve University and The Richard J. Fasenmyer Foundation

Supplemental Figure 3

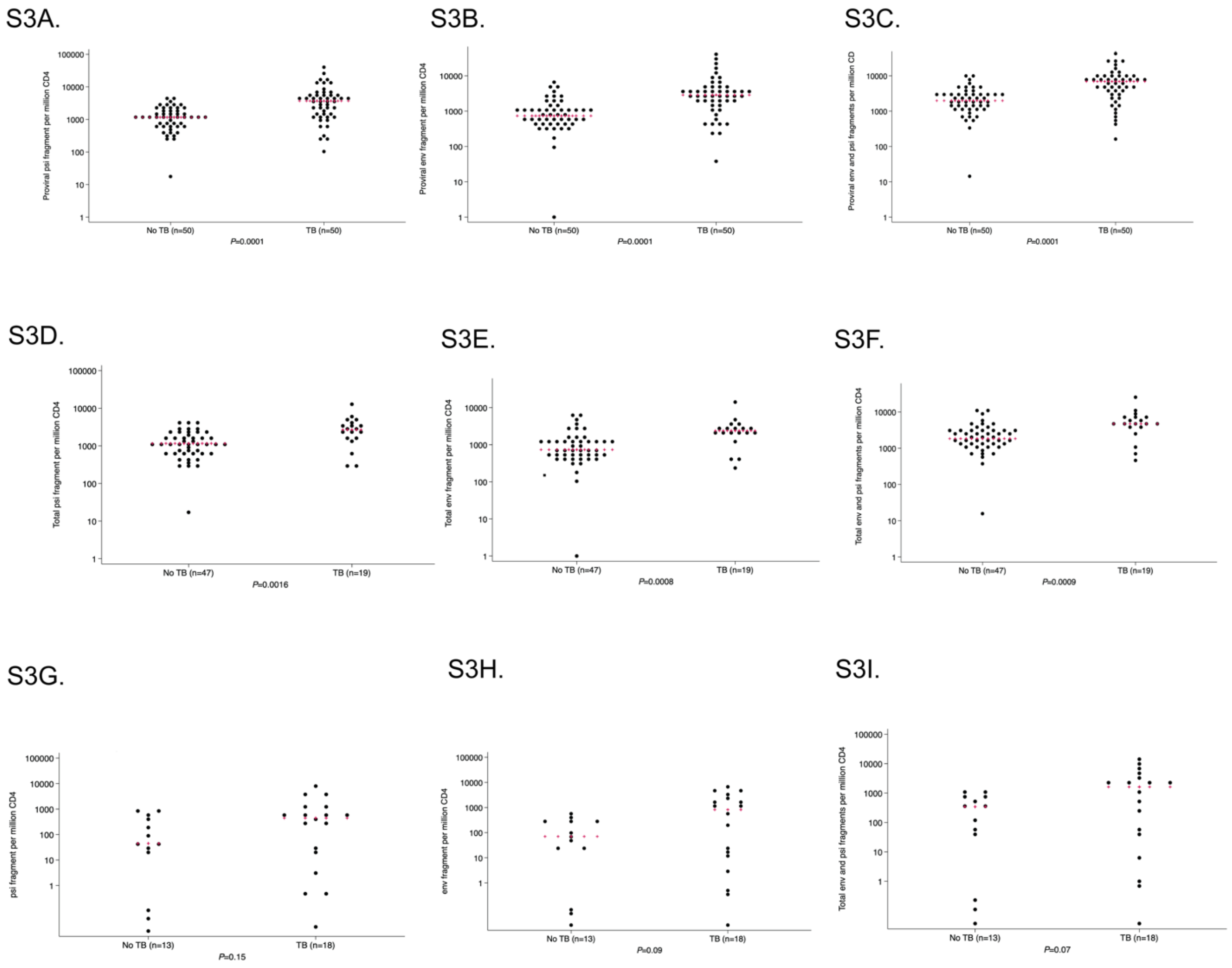

Supplemental Figure 3. Fragment 5' *psi*, fragment 3' *env*, and total non-intact (*psi* + *env)* provirus in the identification cohort (S3A, S3B, S3C, respectively); identification cohort subset (S3C, S3D, S3E, respectively); and validation cohort (S3G, S3H, S3I). Red pluses are the medians. Statistical test was Kruskal-Wallis for non-normally distributed data.

Juste MAJ, Joseph Y, Lespinasse D, Apollon A, Jamshidi P, Lee MH, Ward M, Brill E, Duffus Y, Chukwukere U, Danesh A, Alberto WC, Fitzgerald DW, Pape JW, Jones RB, Dupnik K. People Living With HIV Have More Intact HIV DNA in Circulating CD4+ T Cells if They Have History of Pulmonary Tuberculosis. *Pathogens and Immunity*. 2024;9(2):172–193. doi: 10.20411/pai.v9i2.722
