# Supplementary material for: People Living With HIV Have More Intact HIV DNA in Circulating CD4+ T Cells if They Have History of Pulmonary Tuberculosis
Source: Pathog Immun. 2024 Sep 23;9(2):172–93. doi: 10.20411/pai.v9i2.722 (PMC11432494; doi:10.20411/pai.v9i2.722)
Supplement: Supplemental Methods [file pai-9-172-s02.pdf]

PATHOGENS AND IMMUNITY
IN ASSOCIATION WITH CASE WESTERN RESERVE UNIVERSITY AND THE RICHARD J. FASENMYER FOUNDATION

SUPPLEMENTAL METHODS

1. **Description of parent cohorts for identification and validation studies**
2. **STROBE checklist: TBRU**
3. **STROBE checklist: TB Recurrence**

1. DESCRIPTION OF PARENT COHORTS FOR IDENTIFICATION AND VALIDATION STUDIES

Identification cohort

For the identification cohort, we designed a nested case-control study from a parent cohort study of TB called the Tri-institutional Tuberculosis Research Unit (TBRU) [28-30] (Supplemental Figure 1). For TBRU, adult and adolescent participants were recruited from TB ambulatory care centers at GHESKIO from October 2014 to January 2020. TBRU comprised groups of people with newly diagnosed untreated TB with or without HIV, community controls without history of TB with or without HIV, and a study of families with two or more children with TB in the household. TB was defined as pulmonary TB which had been bacteriologically confirmed by positive sputum TB GeneXpert, positive sputum smear for acid-fast bacilli, or sputum culture that grew *Mycobacterium tuberculosis.* Adult participants with HIV were approached to participate in the study consecutively when they presented to their care provider at GHESKIO for usual care. Participants did not need to be virologically suppressed to be eligible for inclusion in TBRU. Sources of data included participants answering questions using standardized case-report forms and information from the medical record. Latent TB testing was using the Quantiferon-TB Gold In-Tube interferon-gamma release assay (IGRA) (Qiagen, MD, USA). Study data were collected and managed using REDCap electronic data capture tools hosted at Weill Cornell Medicine [31, 32]. Samples were collected for genomics, metabolomics, transcriptomics, and immune studies. Blood for plasma and peripheral blood mononuclear cell (PBMC) isolation was collected with heparin as an anticoagulant.

Juste MAJ, Joseph Y, Lespinasse D, Apollon A, Jamshidi P, Lee MH, Ward M, Brill E, Duffus Y, Chukwukere U, Danesh A, Alberto WC, Fitzgerald DW, Pape JW, Jones RB, Dupnik K. People Living With HIV Have More Intact HIV DNA in Circulating CD4+ T Cells if They Have History of Pulmonary Tuberculosis. *Pathogens and Immunity*. 2024;9(2):172–193. doi: 10.20411/pai.v9i2.722

PATHOGENS AND IMMUNITY
In Association with Case Western Reserve University and The Richard J. Fasenmyer Foundation

The initial sample size for the nested case-control study was selected based on adequate samples and funding to complete IPDA, since there were no data published on variations in IPDA-generated proviral load in people with and without history of TB. The selection criteria for inclusion in the nested case-control study were people age 18 or over with confirmed HIV infection and adequate PBMC aliquots available in New York. Within these criteria, the database manager selected 25 people with a new diagnosis of bacteriologically confirmed pulmonary TB and 25 people with a history of bacteriologically confirmed pulmonary TB and then incidence-density matched controls 1:1 by sex and age to a "no TB" group comprised of 50 participants of whom 25 had positive IGRA and 25 had negative IGRA for TB. Except for the family study, TBRU was a single-visit study with single phlebotomy for biobanking. Participants included in the identification case-control study were recruited and their PBMC cryopreserved between June 2015 and January 2019.

Validation cohort

For the validation study, we used PBMC collected as part of a parent case-control study of TB recurrence in people living with HIV (Supplemental Figure 2). For the TB recurrence parent study, adult participants were enrolled in a study of the human immune response to TB with an emphasis on recurrent TB at GHESKIO from December 2019 to October 2023. Recurrent TB was defined as at least two episodes of bacteriologically confirmed TB, with the second episode occurring at least 6 months after completing treatment for the first episode. Both episodes of TB were to have been diagnosed concurrently with or after diagnosis of HIV. Each episode of TB was bacteriologically confirmed with positive sputum TB GeneXpert, positive sputum smear for acid-fast bacilli, or sputum culture that grew *M. tuberculosis*. TB treatment for each participant was completed at least 6 months before enrollment into the study.

PATHOGENS AND IMMUNITY
In Association with Case Western Reserve University and The Richard J. Fasenmyer Foundation

Participants with history of recurrent TB, one episode of TB, or no episodes of TB were identified by way of a query of the GHESKIO medical record and then invited to participate in the study. All participants had HIV which was virologically suppressed at the last available laboratory assessment of viral load. Participants had one study visit with questionnaire, demographics, and phlebotomy. Sources of data included participants answering questions using standardized case-report forms and information from the medical record. Latent TB testing was using the Quantiferon-TB Gold Plus In-Tube interferon-gamma release assay (IGRA) (Qiagen, MD, USA). Study data were collected and managed using REDCap electronic data capture tools hosted at Weill Cornell Medicine [31, 32]. Blood for plasma and peripheral blood mononuclear cell (PBMC) isolation was collected with EDTA as an anticoagulant. The nested case-control study included all study participants with adequate PBMC available in New York as of October 2021. We calculated that 13 people in the TB group and 13 people in the non-TB group would give 90% power to detect the difference in proviral loads between TB and non-TB groups seen in the identification subcohort with a standard deviation of 500. Study participants were enrolled and PBMCs were collected between December 2019 and October 2020.

PATHOGENS AND IMMUNITY
In Association with Case Western Reserve University and The Richard J. Fasenmyer Foundation

2. STROBE CHECKLIST: TBRU

Identification cohort: Nested case-control study from parent Tri-Institutional Tuberculosis Research Unit (TBRU) study

Title and abstract
1a. The manuscript study's design (case-control) is included in the abstract. The parent TBRU study design (cohort) is indicated in the Methods.
1b. The abstract includes a summary of what was done and what was found.

Introduction
Background/rationale
2. The scientific background and rationale are detailed in the Background
Objectives
3. The objective and hypothesis are included in the Background.

Methods
Study design
4. The study design for the parent study and the case-control study are described early in the Methods, only after description of the study site and study ethical review.
Setting
5. GHESKIO Centers, Port au Prince, Haiti is described in the first paragraph of the Methods. The dates for recruitment and enrollment in the parent study, and for the subset of people in the case-control study, are included in the Methods.
Participants
6a. The eligibility criteria for parent and nested case-control study are included in the Methods section. The rationale for choice of cases and controls is included.
6b. The matching criteria are included. The 1:1 ratio of cases to controls is included.
Variables
7. Criteria for diagnosis of bacteriologically confirmed TB are included. Potential confounders and effect modifiers are addressed in the Results section.
Data sources/measurement
8. We have included statement about sources of data in the Methods section.
Bias
9. We included potential sources of bias in the "Data and statistical analysis" section of the Methods.
Study size
10. We have included a statement regarding the rationale for the sample size of the case-control identification cohort.
Quantitative variables
11. Quantitative variables were maintained as quantitative and not grouped.
Statistical methods
12a. Statistical methods and attempts to control for confounding are detailed.
12b. Methods to describe subgroups (new vs history of TB, IGRA positive vs. IGRA negative) are included.
12c. People with missing plasma samples were excluded from the sub-cohort because we could not assess them for circulating p24 antigen.
12d. Approach for selection of cases and controls is included.
12e. Sensitivity analyses were not completed.

Juste MAJ, Joseph Y, Lespinasse D, Apollon A, Jamshidi P, Lee MH, Ward M, Brill E, Duffus Y, Chukwukere U, Danesh A, Alberto WC, Fitzgerald DW, Pape JW, Jones RB, Dupnik K. People Living With HIV Have More Intact HIV DNA in Circulating CD4+ T Cells if They Have History of Pulmonary Tuberculosis. *Pathogens and Immunity*. 2024;9(2):172–193. doi: 10.20411/pai.v9i2.722

Results
Participants
13a. Numbers of individuals in each study are included in the text and as part of Supplemental Figure 1.
13b. Reasons for non-participation are given and included in Supplemental Figure 1.
13c. A flow diagram is included as Supplemental Figure 1.
Descriptive data
14a. Characteristics of study participants at each stage are included in Tables 1 and 2.
14b. Participants with missing data for p24 were excluded from the case-control analysis group requiring negative plasma p24 for inclusion.
Outcome data
15. Provirus levels are reported as medians with inter-quartile range in the TB and non-TB groups.
Main results
16a. Because of the non-normal distribution of the provirus quantitation, we report medians and inter-quartile ranges.
16b. Continuous variables were not categorized.
16c. Risk estimates are not part of this study.
Other analyses
17. Analyses of subgroups are included in the Results.

Discussion
Key results
18. Key results of intact and total provirus levels are summarized in the TB vs. no-TB groups with considerations for potential confounders and other factors.
Limitations
19. Limitations are elaborated in the discussion section, including potential sources of imprecision.
Interpretation
20. Our results are discussed with consideration for findings in the published literature.
Generalizability
21. Discussion of generalizability of our results are included.

Other information
22. Funding sources for the research and investigators are included in the “Financial Contributions” section of the Addenda.

PATHOGENS AND IMMUNITY
IN ASSOCIATION WITH CASE WESTERN RESERVE UNIVERSITY AND THE RICHARD J. FASENMYER FOUNDATION

3. STROBE CHECKLIST: TB RECURRENCE
Validation cohort: TB Recurrence

Title and abstract
1a. The manuscript study's design (case-control) is included in the abstract. The parent TB Recurrence study design (case-control) is indicated in the Methods.
1b. The abstract includes a summary of what was done and what was found.

Introduction
Background/rationale
2. The scientific background and rationale are detailed in the Background
Objectives
3. The objective and hypothesis are included in the Background.

Methods
Study design
4. The study design for the parent study and the case-control study are described early in the Methods, after description of the study site and study ethical review.
Setting
5. GHESKIO Centers, Port au Prince, Haiti is described in the first paragaph of the Methods. The dates for recruitment and enrollment in the parent study, and for the subset of people in the case-control study, are included in the Methods.
Participants
6a. The eligibility criteria for parent and nested case-control study are included in the Methods section. The rationale for choice of cases and controls is included.
6b. This study used available samples and did not directly match cases to controls.
Variables
7. Criteria for diagnosis of bacteriologically confirmed TB are included. Potential confounders and effect modifiers are addressed in the Results section.
Data sources/measurement
8. We have included statement about sources of data in the Methods section.
Bias
9. We included potential sources of bias in the "Data and statistical analysis" section of the Methods.
Study size
10. We have included a statement regarding the sample size calculation for the validation cohort.
Quantitative variables
11. Quantitative variables were maintained as quantitative and not grouped.
Statistical methods
12a. Statistical methods and attempts to control for confounding are detailed.
12b. Methods to describe subgroups, including recurrent vs. single episode of TB) are included.
12c. People with positive viral load and p24 were excluded because the viremia could impact the assessment of reservoir with the IPDA.
12d. Approach for selection of cases and controls is included.
12e. Sensitivity analyses were not completed.

Results

Participants

13a. Numbers of individuals in each study are included in the text and as part of Supplemental Figure 2.
13b. Reasons for non-participation are given and included in Supplemental Figure 2.
13c. A flow diagram is included as Supplemental Figure 2.

Descriptive data

14a. Characteristics of study participants at each stage are included in Table 3.
14b. Participants with missing data for p24 were excluded from the case-control analysis group requiring negative plasma p24 for inclusion.

Outcome data

15. Provirus levels are reported as medians with inter-quartile range in the TB and non-TB groups.

Main results

16a. Because of the non-normal distribution of the provirus quantitation, we report medians and inter-quartile ranges.
16b. Continuous variables were not categorized.
16c. Risk estimates are not part of this study.

Other analyses

17. Analyses of subgroups are included in the Results.

Discussion

Key results

18. Key results of intact and total provirus levels are summarized in the TB vs. no-TB groups with considerations for potential confounders and other factors.

Limitations

19. Limitations are elaborated in the discussion section, including potential sources of imprecision.

Interpretation

20. Our results are discussed with consideration for findings in the published literature.

Generalisability

21. Discussion of generalizability of our results are included.

Other information

22. Funding sources for the research and investigators are included in the "Financial Contributions" section of the Addenda.
